# The Multidisciplinary Management of Perianal Fistulas in Crohn’s Disease: A Systematic Review

**DOI:** 10.7759/cureus.29347

**Published:** 2022-09-20

**Authors:** Omar Badla, Raman Goit, Samia E Saddik, Sarah Dawood, Ahmad M Rabih, Ahmad Mohammed, Aishwarya Raman, Manish Uprety, Maria Calero, Maria Resah B Villanueva, Narges Joshaghani, Nicole Villa, Lubna Mohammed

**Affiliations:** 1 General Surgery, California Institute of Behavioral Neurosciences & Psychology, Fairfield, USA; 2 Internal Medicine, California Institute of Behavioral Neurosciences & Psychology, Fairfield, USA; 3 Pediatrics, California Institute of Behavioral Neurosciences & Psychology, Fairfield, USA; 4 Obstetrics and Gynecology, California Institute of Behavioral Neurosciences & Psychology, Fairfield, USA; 5 Research, California Institute of Behavioral Neurosciences & Psychology, Fairfield, USA; 6 Psychiatry and Behavioral Sciences, California Institute of Behavioral Neurosciences & Psychology, Fairfield, USA

**Keywords:** perianal fistula, medical and surgical management, treatment options of perianal fistula in crohn’s disease, perianal fistula in crohn’s disease, crohn’s disease (cd)

## Abstract

Perianal fistulas in Crohn's disease (CD) are often recurring and challenging to treat. This systematic review aimed to evaluate the medical, surgical, and combination treatment options and provide an overview of their efficacy. We performed this systematic review following the Preferred Reporting Items for Systematic reviews and Meta-Analyses (PRISMA) guidelines. Our group searched PubMed, Medline, PubMed Central, Google Scholar, and ScienceDirect for articles within the last ten years using different terms and criteria mentioned in detail in the search strategy and eligibility criteria sections. Initially, 739 records were retrieved, out of which we excluded 731 records for various reasons, such as irrelevant titles and abstracts and low scores on quality assessment tools. The evidence for combination (surgical and medical) therapy is superior to that for medical and surgical treatments individually. In contrast, the studies on medical and surgical treatments individually reported varied evidence and efficacy for their respective options.

## Introduction and background

Crohn's disease (CD) is a chronic disease characterized by transmural inflammation, which can involve the entirety of the gastrointestinal tract from mouth to anus [1]. According to the Montreal Classification, the CD is classified into four categories: inflammatory, stricturing, penetrating, and perianal disease [2]. Perianal fistulas, defined as an abnormal connection between a portion of the intestine and the anorectum and skin of the buttock, arise in the setting of penetrating CD [1]. Perianal fistulas were recognized as complications of CD by Penner and Crohn in the year 1939 [3]. This complication occurs in almost one-third of patients with CD within the first twenty years of diagnosis [4]. The pathogenesis of perianal fistulas in CD has not been fully elucidated, but it is thought to involve a combination of inflammatory, genetic, bacterial, and immune processes [5,6]. In addition, patients with long-standing, early-onset CD involving the rectum and colon are more likely to develop perianal fistulas [3,6,7].

Perianal fistulas are a source of additional morbidity in CD patients. They can lead to the following symptoms: localized pain, discharge, and local tissue destruction, eventually leading to sphincter dysfunction [8]. In addition to the dysfunction caused by the symptoms mentioned, perianal fistulas in CD recur in about one-third of patients, significantly increasing morbidity. That being said, many patients who attain healing sustain long remission terms [9]. Unfortunately, the recurring nature of perianal fistulas in CD makes them challenging to treat, often requiring a multidisciplinary approach in which a surgeon, a gastroenterologist, and a radiologist are involved [5]. The two primary goals when treating perianal fistulas in CD are to attain healing of the fistula tract and prevent and treat infection and sepsis [6]. A typical treatment regimen includes medical interventions such as antibiotics, immunosuppressants (e.g., azathioprine and mercaptopurines), anti-tumor necrosis factor (TNF) drugs, and surgical interventions such as anal fistula plug, a draining seton, fistulotomy, stoma formation and proctectomy [5,10].

Despite all these treatment options, healthcare providers still face several issues when attempting to treat perianal fistulas in CD. The effectiveness of medical therapy is limited as almost two-thirds of patients relapse after its cessation [4]. In addition, the use of anti-TNF drugs is associated with side effects such as headache, fatigue, and increased risk of infections making their use long-term a potentially unsafe practice [8]. Surgical management has various drawbacks, including anal incontinence, relapse, and inadequate healing [4]. A portion of patients treated surgically often require a total proctectomy, further showcasing the refractory nature of this condition [4]. Anti-integrins, interleukin 12/23 pathway inhibitors, and stem cell therapies are potential treatment options, but their incorporation into routine practice remains uncertain [4,6]. In the surgical realm, newer procedures that have emerged include the ligation of inter-sphincteric fistula tract (LIFT) procedure, the video-assisted anal fistula treatment (VAAFT), and the over-the-scope clip (OTSC) [10,11].

Due to the challenging aspects of maintaining remission in these patients and the emergence of newer drugs and procedures with differing levels of evidence supporting them, there is still no agreed-upon high-quality treatment regimen for perianal fistulas in CD. In light of this, we performed a systematic review to assess the various treatment options available and provide an overview of the current evidence.

Methods

We performed This systematic review in accordance with the Preferred Reporting Items for Systematic reviews and Meta-Analyses (PRISMA) guidelines [12].

Eligibility Criteria

The characteristics of the studies we searched were: Full-text papers, papers published between 2012 and 2022, studies performed on human subjects, studies in the English language, study designs including meta-analyses, clinical trials, randomized control trials, systematic reviews, and case reports.

Search Strategy

In order to gather articles relevant to the topic, two reviewers thoroughly searched the following databases (PubMed, Medline, PubMed Central, Google Scholar, and ScienceDirect). We performed the last search on April 14, 2022. Table 1 outlines the details of the search strategy, including filters, terms used, and databases searched. 

**Table 1:** Details of the search strategy Mesh: Medical Subject Headings

Databases	Mesh and other keywords searched	Filters	Number of results
PubMed, Medline, PubMed Central	Crohn’s disease OR Crohn disease OR ( "Crohn Disease/complications"[Mesh] AND "Crohn Disease/drug therapy"[Mesh] AND "Crohn Disease/surgery"[Mesh] OR "Crohn Disease/therapy"[Mesh] ) AND Rectal Fistula OR ( "Rectal Fistula/drug therapy"[Mesh] AND "Rectal Fistula/surgery"[Mesh] OR "Rectal Fistula/therapy"[Mesh] )	Full Text, Clinical Trial, Meta-Analysis, Systematic Review, Randomized Controlled Trial, Review, 2012-2022, Humans, English	389
Science Direct	Fistulizing Crohns treatment	2012-2022	Screened the first 50 results
Google Scholar	Crohns fistula treatment	2012-2022	Screened the first 300 results

Quality Assessment

Table 2 summarizes the quality assessment process which yielded the final eight studies.

**Table 2:** The studies which passed the quality assessment and what tools were used PRISMA: Preferred Reporting Items for Systematic Reviews and Meta-Analyses, SANRA: Scale for the Assessment of Narrative Review Articles

Quality assessment tool	Study design	The maximum score that can be attained	Score accepted (more than 70%)	Studies with passing scores
PRISMA [12]	Systematic reviews and meta-analyses	27	19	Yassin et al. [5], De Groof et al. [11], Braithwaite et al. [1], Lee et al. [10], Fu et al. [13], Lee et al. [6], Cheng et al. [4].
SANRA [14]	Traditional review	12	9	Klag et al. [7].

Results

**Figure 1:**
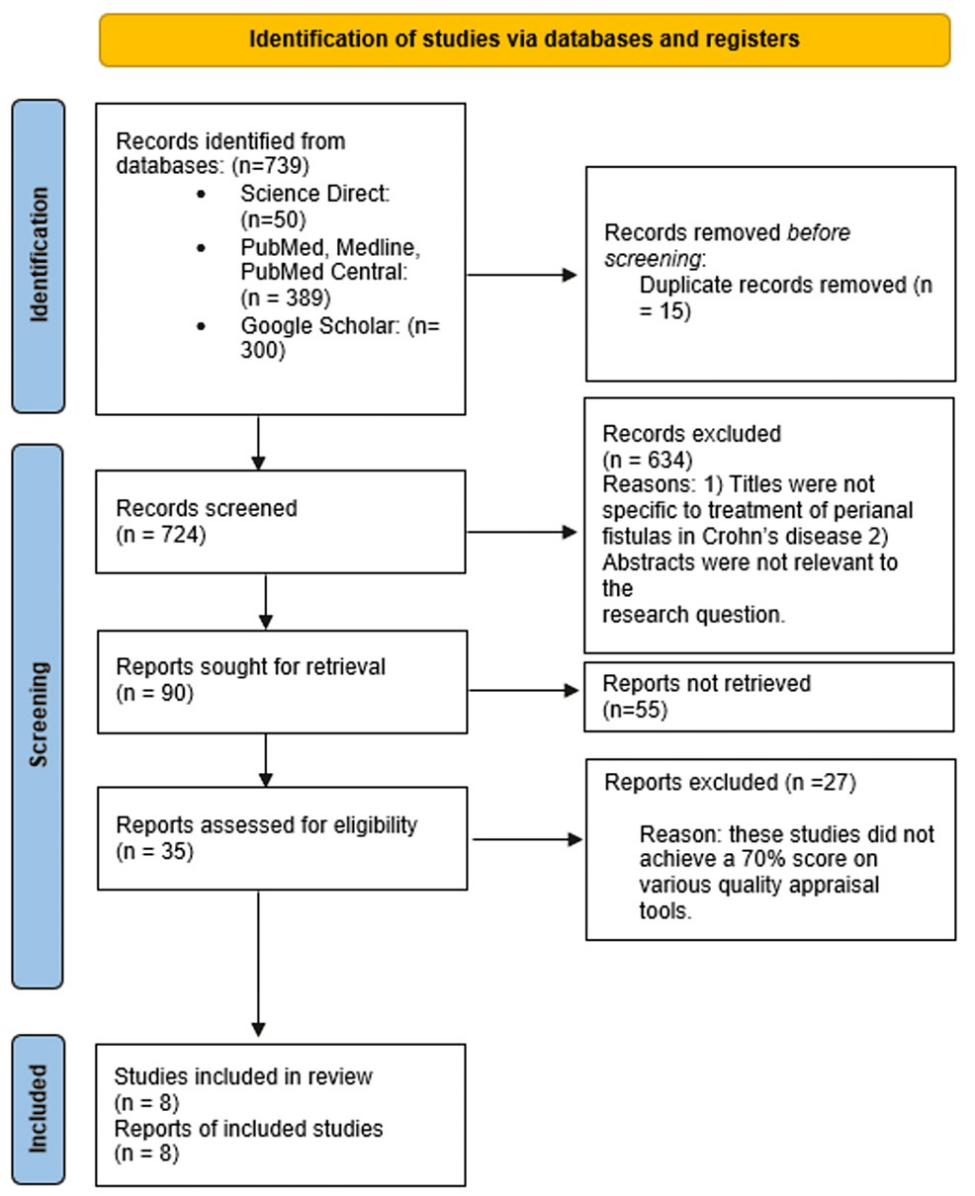
PRISMA 2020 flow diagram for new systematic reviews depicting the study selection process PRISMA: Preferred Reporting Items for Systematic Reviews and Meta-Analyses

Seven hundred thirty-nine records were identified from three databases: PubMed, Medline, Pubmed Central, Google Scholar, and ScienceDirect. We screened the first 300 articles from Google Scholar, the first 50 articles from ScienceDirect, and all 389 articles from PubMed, Medline, and PubMed Central, and we removed 15 articles as they were duplicates. In addition, we excluded 634 more articles for various reasons. Firstly, we removed articles with titles that did not include perianal fistulas on CD. Secondly, we removed articles with abstracts that focused on topics other than the treatment of perianal fistulas in CD. Thirdly, we excluded articles initially written in languages other than English. Finally, two independent reviewers assessed the remaining 35 articles for eligibility and excluded 27 as they did not achieve a 70% score on their respective quality assessment tools. After this process, we chose eight final articles and included them in this systematic review. This process is outlined in Figure 1.

This study investigates multiple treatment modalities within the medical and surgical disciplines, and naturally, the effectiveness of and evidence supporting these treatment options vary. Table 3 presents the conclusions and characteristics of the primary studies of this systematic review.

**Table 3:** The characteristics and conclusions of the eight studies included in this systematic review. MSC: Mesenchymal stem-cell, CD: Crohn's Disease, TNF: Tumor necrosis factor

Author	Year	Study Design	Modality of Treatment/Factors Investigated	Conclusion
Cheng et al. [4]	2019	Systematic review and meta-analysis	MSC therapy	This treatment modality has shown promising results in terms of safety and efficacy in treating perianal fistulas in CD.
Lee et al. [6]	2018	Systematic review and meta-analysis	Medical therapies	Anti-TNF drugs were successful at inducing and maintaining response and remission for perianal fistulas in CD.
Fu et al. [13]	2017	Meta-analysis	Adalimumab	Adalimumab is safe and efficacious in patients with perianal fistulas in CD that are no longer sensitive to infliximab or patients who have not been treated with biologics previously.
Lee et al. [10]	2017	Systematic review	Surgical interventions	This study formulated no specific conclusions due to clinical heterogeneity and bias in the studies it evaluated.
Braithwaite et al. [1]	2017	Systematic review	Prognostic factors	This study shed some light on potential prognostic factors that may guide treatment. However, there is a need for a prospective cohort with well-defined patient characteristics to draw definite conclusions.
De Groof et al. [11]	2016	Systematic review	Surgical and medical treatment guidelines	The conclusions of this study varied vastly in quality of evidence and are detailed in the discussion section.
Klag et al. [7]	2015	Traditional review	Medical therapy	Several medical treatment options such as antimicrobials, immunosuppressants, and anti-TNF drugs have shown a beneficial role in treating perianal fistulas in CD.
Yassin et al. [5]	2014	Systematic review	Combination of medical and surgical treatment	Combined therapy has shown superior efficacy to either of the treatment modalities alone.

## Review

Discussion

The treatment of perianal fistulas in CD is complex and requires a multidisciplinary team; therefore, the discussion will focus on exploring the various treatment options within medical, surgical, or combination regimens.

Medical Treatments

Aminosalicylates and corticosteroids: When used to treat perianal fistulas in CD, corticosteroids have shown no efficacy in improving the disease and have also increased discharge and the number of surgical consultations [10]. Similarly, aminosalicylates have not been shown to improve perianal fistulas in CD [10].

Anti-tumor necrosis factor drugs: Out of all medical therapies, this class of drugs has the most convincing statistics supporting its role in treating perianal fistulas in CD. In a meta-analysis evaluating the efficacy of medical therapies in perianal fistulas in CD, anti-TNF drugs have been shown to increase the rate of therapeutic induction by 1.5 times and double the rate of remission and maintenance of therapeutic response [6]. Data comparing infliximab and adalimumab are limited, but a meta-analysis evaluating the efficacy of adalimumab draws similarities between the mechanism of action of both drugs, their effectiveness, and safety but states that infliximab is more likely to cause allergic phenomena [6,13]. A systematic review analyzing the evidence of national and international guidelines for perianal fistulas further supports the role of both drugs with a level of evidence 1a (highest level) [11]. A study assessing the routine use of endoscopic ultrasounds to guide medical decisions in patients treated with adalimumab has shown some clinical benefits [15]. During the first 24 weeks, patients in the intervention arm experienced a faster amelioration of symptoms. This faster rate was due to the endoscopic ultrasound's ability to detect subclinical worsening of disease activity and subsequent dose increases in adalimumab [15]. Another benefit of this intervention is that it could detect an early need for surgical re-intervention [15]. A limitation of this study is that the sample size was too small, and a larger study could provide more robust evidence for incorporating this intervention [15]. Certolizumab is another anti-TNF drug that has been tested for fistulas in CD, but the results are inconclusive. Some results point to no noteworthy enhancement in the closure of fistulas, and other results show that it may be effective in healing fistulas in those who have previously responded to it [7]. The minimum serum concentrations of infliximab and adalimumab that are considered therapeutic for fistula closure are 5.0 µg/mL and 5.9 µg/mL, respectively [16]. One of the limitations of the role of this class in treating perianal fistulas in CD is the high rate of relapse after discontinuation. Up to 50% of patients who discontinue the drug will suffer from relapse within five years [17]. With that in mind, long-term administration becomes necessary to maintain remission. However, with this class's potentially severe side effects (increased risk of infection), other avenues have to be explored to achieve this goal [8]. One of those avenues is to use combinations of this class with other classes, which we will explore in the coming sections.

Antimicrobials: The role of antimicrobials (ciprofloxacin and metronidazole) in the treatment of perianal fistulas in CD contributes to improving the fistula itself and managing septic complications that accompany it [3,7,11]. However, no unanimity has been reached within the guidelines on whether they should be used as first or second-line agents [11]. The evidence shows that when used alone, antimicrobials' efficacy is limited and not recommended by the AGA clinical practice guidelines on the medical management of moderate to severe luminal and perianal fistulizing CD [2,7]. Therefore, the role of this class is prominent only when used as an adjunct to biologics and thiopurines [3,11]. Combining ciprofloxacin and adalimumab has proved superior in efficacy to adalimumab alone in inducing and maintaining remission [6,7]. Another combination that involves antibiotics is its use with thiopurines. Antibiotics are only used initially in this combination and then discontinued, but thiopurines' continued use sustains the effect [7]. Indications for the usage of antibiotics are not well defined. However, a study evaluating prognostic factors that affect the outcome of perianal fistulas in CD has shown that carriers of a mutated NOD2/CARD15 are less likely to respond to antibiotics. However, this study had a small number of participants with the mutated variant indicating the need for a larger study with similar results to consider this variation when managing patients [1].

Thiopurines: The evidence supporting thiopurines is lacking, and it points to them either having a moderate or no effect on perianal fistulas in CD when used alone [3,6]. Most guidelines are in agreement that cyclosporine and methotrexate have poor efficacy. Oral tacrolimus is the only drug in this class that has shown possible efficacy in inducing a therapeutic response. However, due to its adverse effects, healthcare providers have to be cautious by surveilling the serum concentrations [3,6,11].

Ustekinumab:* *Ustekinumab is a monoclonal antibody that exerts its action by antagonizing the shared p40 subunit of interleukins 12 and 23 [6]. Promising data has emerged regarding the potential incorporation of this drug in treatment regimens for perianal fistulas in CD. Data pooled from studies in phases one to three on ustekinumab's efficacy reveals that the drug is associated with a higher rate (1.5 times) of therapeutic response induction in perianal fistulas in CD compared to placebo. As these studies are not in phase four yet, this data should be viewed as experimental, and the statistics are from post-hoc analyses [6]. Another study conducted on patients on CD patients with perianal fistulas whose symptoms were resistant to anti-TNF drugs revealed that ustekinumab was effective. However, this study's small number of participants limited its validity [18]. In conclusion, researchers should investigate this drug in a larger number of patients to provide higher quality evidence [9].

Vedolizumab: This drug is a monoclonal antibody that targets a4b7 integrin, which blocks the migration of T-cells into the gastrointestinal tissue [6]. Much like ustekinumab, this drug shows promise, but the results are still considered experimental [6]. A randomized controlled trial that evaluated a subgroup of patients suffering from perianal fistulas in CD stated that the drug might be better than the placebo for symptom amelioration. However, the evidence was deemed insufficient due to multiple biases [2]. An exploratory analysis of the GEMINI 2 trial also concluded that vedolizumab could have a role in treating perianal fistulas in CD, but that is subject to confirmation by prospective clinical studies devoted to this topic [19].

Surgical Treatments

Abscess drainage: Abscess drainage is commonly used in practice by physicians, and it aims to facilitate drainage of perianal abscesses, which usually coexist with perianal fistulas in CD. The goal of this intervention is to decrease the risk of septic complications which may be enhanced by administering immunomodulatory drugs [3].

Setons:* *The most supported use of this treatment modality is to facilitate long-term drainage and promote healing [3,5]. One shortcoming pertaining to the use of setons is that they block the process of complete closure of the fistula tract; hence, their use is mostly palliative and requires keeping the seton in situ [3,5,10]. In addition, the duration for which the seton should remain in situ has not been clearly established. In one study, removal after two weeks resulted in a recurrence rate of 15%. In contrast, another study reported keeping the seton for the period when infliximab was used for induction, and the recurrence rate was 0% [3]. Due to the lower risk of external sphincter damage and subsequent incontinence, loose setons are favored over cutting setons [3,11]. In conclusion, the use of setons is optimal when kept for the duration of induction with infliximab. Furthermore, clinicians prefer it as it can conservatively prevent septic episodes without significant risk of damaging the sphincter [3,10].

**Figure 2:**
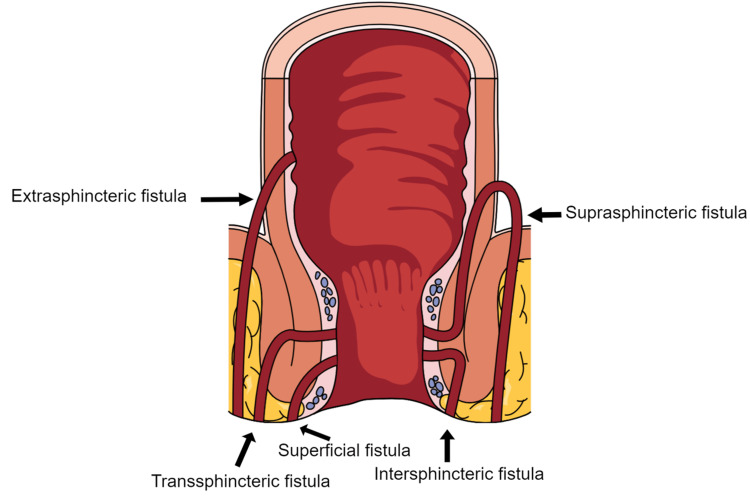
Illustration of fistula classification Figure created by the first author on the ‘’Mind the Graph’’ website

Fistulotomy:* *This procedure has the potential to cause incontinence; therefore, it is used cautiously and reserved for symptomatic, superficial, low (inter-sphincteric/chosen trans-sphincteric) fistulas where the risk of incontinence is low [3,10]. Generally speaking, surgeons should avoid this procedure in patients with high fistulas as the risk of incontinence is significant [11]. Figure 2 depicts the anatomical outline of different types of fistulas.

Advancement flap: This technique aims to cover the internal fistula orifice with a flap derived from rectal mucosa. The goal is to close the fistula while sparing the sphincter system [3]. It is recommended that this procedure is done in tandem with the administration of immunosuppressants/anti-TNF drugs for the closure of high and complex fistulas [5,11,20]. This procedure was reported to successfully close complex fistulas in CD in 60%-64% of cases, with a 9.4% reported rate of incontinence [3,11]. However, only half the patients required no additional repeated procedure; therefore, these numbers must be taken with a pinch of salt [3]. In addition, it is not possible to deploy advancement flaps when the rectum is substantially fibrosed or if there is concurrent proctitis [10].

Ligation of inter-sphincteric fistula tract: This surgical procedure ligates the mature, fibrotic tract of trans-sphincteric fistulas [3]. The studies cited have provided mixed results on this treatment's efficacy; a systematic review of guidelines states that it could be an option for complex fistulas in a subset of patients as the data is still at an early stage [11]. A systematic review of the surgical treatments of anal fistulas in CD cited a prospective cohort where nine out of 15 patients experienced fistula healing, with eight patients sustaining healing after a year [10]. Another systematic review that evaluated medical and surgical treatment of perianal fistulas in CD stated that this procedure is not recommended due to failure of healing [5]. The difference in opinions and small size of the studies necessitate larger studies to ascertain this procedure's efficacy truly.

Stem cell therapy: Due to their effectiveness in regulating immune cells, mesenchymal stem cells (MSC) have been considered a potential treatment option for perianal fistulas in CD [3]. A systematic review and meta-analysis conducted in 2019 on the effectiveness of MSC in the management of perianal fistulas in CD yielded some promising results. The study revealed that autologous, adipose MSCs effectively attained healing clinically. This treatment is used in patients whose disease course has been refractory to anti-TNF drugs [4]. A study that compared three different MSCs doses with placebo concluded that a dose of 3 x 107 MSCs was the one that had the best effect on healing, and the process caused no significant side effects [21]. The long-term sustainability of the effect of adipose-derived MSCs has also been evaluated, and the results were encouraging [22]. One study evaluating the efficacy of allogeneic bone marrow stem MSCs in perianal fistulas in CD has shown that fistulas that attained closure a month into therapy maintained it four years after [23].

Plugs: A systematic review assessing surgical treatments for perianal fistulas in CD found a significant variation in the rates of favorable outcomes after fistula plug use, stretching from 15% to 86% [10]. Furthermore, complications such as abscess formation, sepsis, and failure rates have restricted the role of this procedure [10]. Another systematic review supported this notion and cited poor healing rates as the cause [5]. A systematic review of the guidelines had differing views and concluded that the use of plugs is reasonable, but the emphasis was put on the need for further research [11]. A randomized controlled trial that compared the use of plugs to the removal of setons found that plugs were not superior at achieving closure [24].

Fibrin glue: A consensus has not been reached on the use of this modality. Two systematic reviews state that glues have either become obsolete or were not recommended in the first place, citing poor healing as a cause [5,10]. A systematic review of guidelines states that it is possible that surgeons can use this modality for complex perianal fistulas in CD, but the evidence is considered unclear, and more research is required [11]. A retrospective study supports this notion and states that a role as an adjunct to medical treatment may be efficacious [25].

Video-Assisted Anal Fistula Treatment (VAAFT)

VAAFT is a relatively newer surgical technique that treats anal fistulas by attempting to achieve three goals. Firstly, a fistuloscope is inserted into the main track's outer orifice to identify and electrocauterize secondary tracks. Secondly, the inner orifice is sealed utilizing an advancement flap. Thirdly, removal of the main track is done when plausible [10]. Data on this modality were scarce and involved a prospective study that reported on 11 patients with anal fistulas in the setting of CD who were treated with a combination of VAAFT, advancement flap, and fecal diversion. Nine out of the 11 surgeries were successful; however, the data did not report long-term maintenance rates. Also, one of the 11 patients had a rectovaginal fistula, further diluting the number of perianal fistulas in this study. The small number of perianal fistulas in CD patients reported signals the need for larger studies using VAAFT to assess its efficacy in perianal fistulas in CD [10].

Over-the-scope clip (OTSC): OTSC is another recently introduced procedure, which accounts for data sparsity on its use. For example, one case series reported on the use of this technique in 10 patients, six of whom had Crohn's trans-sphincteric fistulas. The study reported that all but one of the patients had successful results after a mean follow-up time of 230.5 days [10]. This data, however, need support as it is only performed on a small number of patients and does not report long-term recurrence rates.

Stoma and proctectomy: These two procedures are often interlinked when it comes to the treatment of perianal fistulas in CD. Their use is usually restricted to highly complex and severe cases of fistulizing CD [11]. It is not easy to ascertain what indications signal the need for these procedures exactly. However, the presence of proctitis has been linked with an increased need for proctectomy, indicating that it could be a helpful predictor [1]. The creation of a stoma is implemented in complicated cases but is limited by the inability to regain bowel cohesion which necessitates a follow-up proctectomy [11]. Surgeons reserve the implementation of a proctectomy for cases refractory to conventional medical and surgical therapies [11].

Combined therapy: The presence of many treatment options with varying combinations and evidence levels supporting their use is one of the major roadblocks in formulating a concrete algorithm for managing perianal fistulas in CD. However, the evidence favors a combined medical and surgical regimen over only a medical or surgical regimen alone [5,11]. For example, a study comparing the percentage of healing in patients undergoing surgery alone vs. patients undergoing surgery and receiving an anti-TNF drug revealed a difference of 35% in favor of the combination group (36% vs. 71%) [26]. Another study had similar findings when comparing a group who received anti-TNF drugs and a seton vs. a group who received anti-TNF therapy alone, with the healing percentages again favoring the combination group (63% vs. 75%) [27].

Limitations

This study is limited by the lack of data comparing individual surgical treatment modalities to each other and a lack of high-quality evidence for some of the surgical techniques. Moreover, specific indications for the use of surgical procedures are poorly defined. The conclusions for medical treatments were more definitive than those of surgical treatments, with studies having more decisive statements on individual options such as anti-TNF drugs, antimicrobials, and thiopurines. On the other hand, this was not the case for evidence on ustekinumab and vedolizumab, where the need for larger studies was highlighted to support their efficacy. Like many researchers investigating this topic, we faced the issue of clinical heterogeneity. It can often be challenging to select a cohort of patients with similar demographics, disease severity, and type of fistula. There were also differences in outcome measures and time of follow-up. The studies on combination therapies focused on the setons and anti-TNF drugs and did not incorporate other combinations of the many available treatment options.

## Conclusions

In conclusion, the treatment for perianal fistulas in CD remains challenging for physicians due to the availability of a plethora of treatment modalities with a mixed level of evidence supporting them. From medical treatment options, anti-TNF drugs were supported and agreed upon as either a monotherapy or adjunct to other medical or surgical treatments. Antimicrobials have an essential role in sepsis management and as an adjunct to anti-TNF drugs or thiopurines. Ustekinumab and vedolizumab have shown some promise, but larger studies are needed to establish their use truly. Surgical modalities with the most established and agreed upon use include abscess drainage to prevent sepsis and setons as a palliative option. Fistulotomy carries a risk of incontinence; hence, it should be implemented with caution in those with an elevated risk of this complication. Advancement flap use is recommended when used with immunotherapy/anti-TNF drugs, but its use is not possible when the fistula tract is fibrotic, or there is proctitis. OTSC, VAAFT, and LIFT require further investigation. Complications and a lack of supporting evidence limit the use of plugs and glue. Stem cell therapy has emerged more recently and has shown promise in treating cases not responsive to anti-TNF drugs with evidence of sound healing, tolerability, and long-term healing maintenance. Surgeons reserve the use of Stoma and proctectomy as a last resort for complex refractory cases. As the evidence favors combining medical and surgical treatments, the approach should focus on incorporating both gastroenterologists and surgeons. In order to do so optimally, future studies should focus on identifying which combination is the most efficacious and tolerable. Surgical modalities need to be directly compared, perhaps as part of a meta-analysis, to identify the most superior modality to incorporate into a combination regimen. Lastly, the modalities with insufficient evidence need to be further researched with more extensive studies. This study can serve as a reference point for future research as it summarizes the available evidence on various surgical, medical, and combination treatments. In addition, researchers can hopefully use this study to gain insight into what treatments are recommended and used and what treatments have limitations and require further investigation.
